# Supplementary material for: Multi-modal omics analysis of a paediatric melanoma highlights mechanisms underlying treatment resistance
Source: Commun Med (Lond). 2025 Oct 30;5:448. doi: 10.1038/s43856-025-01201-1 (PMC12575648; doi:10.1038/s43856-025-01201-1)
Supplement: Supplementary file 2 — Supplementary Information [file 43856_2025_1201_MOESM2_ESM.pdf]

Supplementary Figure 1. Related to Figure 2. Expression of *MITF, BRAF* and *NRAS* in primary tumour, metastasis and tumour recurrence tissues.

a. Immunofluorescent images from GeoMx DSP. Two sections of the primary tumour (images 1 and 2), lymph node metastases (image 3), healthy lymph node (image 4) and recurrence (image 5) were stained with the melanoma morphology marker kit from Nanostring: S100B (melanoma, green), CD45 (immune cells, red) and Cyto13 (DNA, blue). Circles represent all collected ROIs and their size and location within each tissue slice. ROIs marked with arrows represent healthy tissues.

b. Uniform Manifold Approximation Projection (UMAP) visualisation of the gene expression data from all ROIs from lymph nodes (healthy and metastasis), primary tumour, recurrence, and healthy skin.

c. Bubble plots of *MITF* (left) *and BRAF* (right) gene expression (Q3 normalised counts) represented as bubble size, overlayed on top of images from GeoMx DSP. Images are numbered as in panel a.

Supplementary Figure 2. Related to Figure 4. Increased *VEGFA* expression comes from melanoma cells.

a. Heatmap representation of non-hierarchical clustering of GeoMx ROIs based on log_2(normalised counts) expression of *VEGFA* and *PECAM1*.

b. Representative immunofluorescence images of ROIs A05-A07 and B02 from primary tumour section stained with the melanoma morphology marker kit from Nanostring: S100B (melanoma, green), CD45 (immune cells, red) and Cyto13 (DNA, blue).

c. UMAPs (Feature plots) showing scaled expression of *VEGFA* and *PECAM1* in the snRNA-Seq dataset.

d. Dot plots of scaled expression of *VEGFA* and *PECAM1* in the cell types identified in the snRNA-Seq dataset.

Supplementary Figure 3. Related to Figure 5. Transcriptional heterogeneity of the primary tumour.

a. UMAP of malignant cells coloured by their assignment to melanoma cell states[16]. Bar plot showing the absolute number of malignant cells assigned to each melanoma cell state.

b. Bar plot showing the absolute number of malignant cells assigned to each melanoma cell state.

Supplementary Figure 4. Immune cell landscape of the primary tumour.

a. UMAP visualisation of all immune cells (n = 353) within the primary tumour, coloured by cell types identified with *SingleR*.

b. UMAPs (Feature plots) showing scaled expression of M1 macrophage markers *(HLA-DRA* and *HLA-DRB1)* in the snRNA-Seq immune cell dataset.

c. UMAPs (Feature plots) showing scaled expression of M2 macrophage markers *(CD163* and *MRC1)* in the snRNA-Seq immune cell dataset.

d. UMAP of projection of T cells from this study onto a reference T cell dataset[17]. Black lines denote density of the mapped T cells from this study.

e. Bar plot showing the absolute numbers of T cells assigned to each reference T cell type.

f. Heatmap of normalised expression of immune checkpoint genes in the snRNA-Seq data grouped per Seurat cluster.

g. Heatmap of normalised expression of immune checkpoint genes in the snRNA-Seq data grouped per cell type.

h. Non-hierarchical clustering of expression (log_2(normalized counts)) of immune checkpoint genes derived from the GeoMx ROIs.

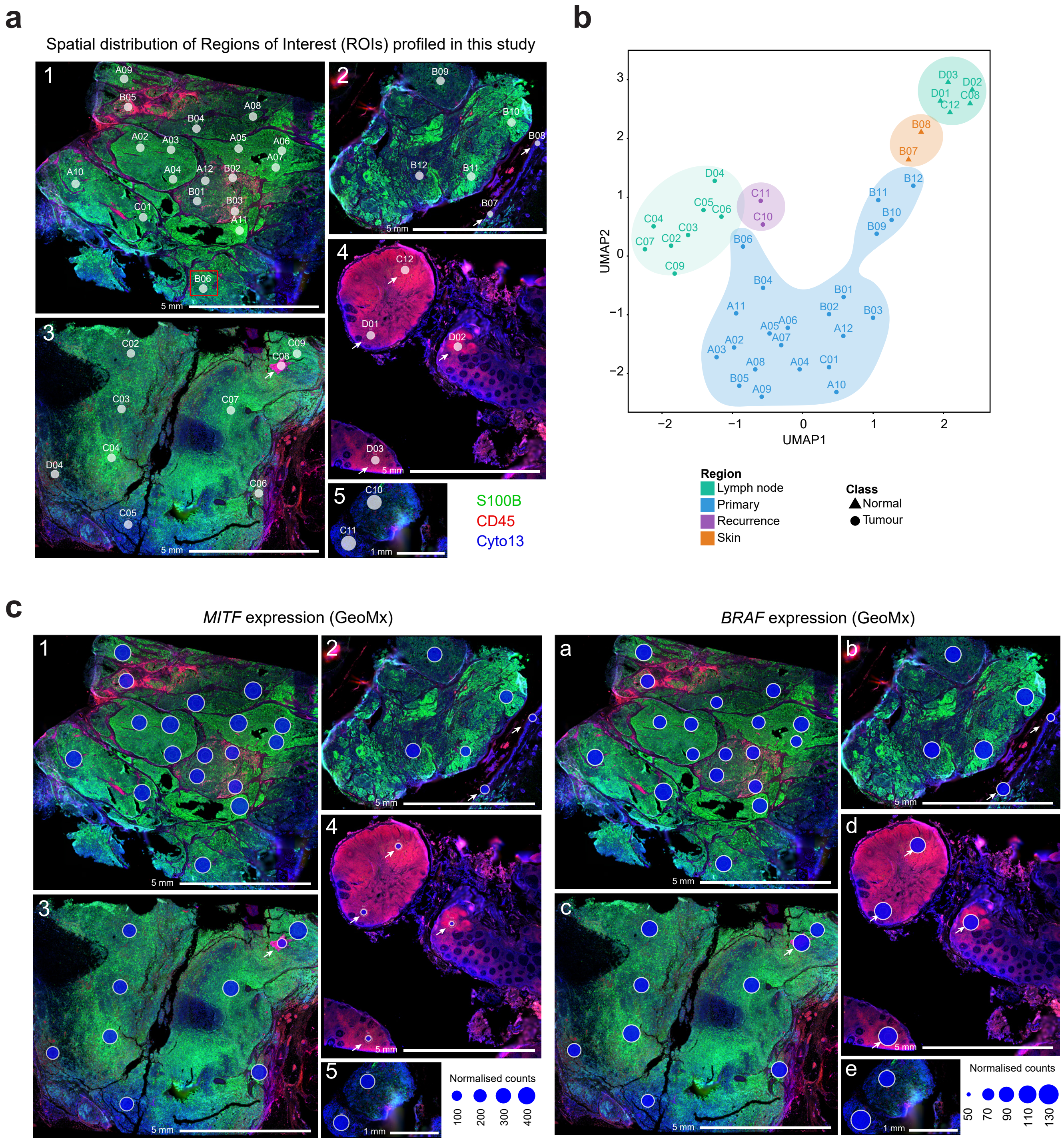
a
Spatial distribution of Regions of Interest (ROIs) profiled in this study
1
2
3
4
5
5 mm
1 mm
S100B
CD45
Cyto13
b
UMAP1
UMAP2
−2
−1
0
1
2
3
Region
Lymph node
Primary
Recurrence
Skin
Class
Normal
Tumour
c
MITF expression (GeoMx)
BRAF expression (GeoMx)
Normalised counts
100
200
300
400
50
70
90
110
130

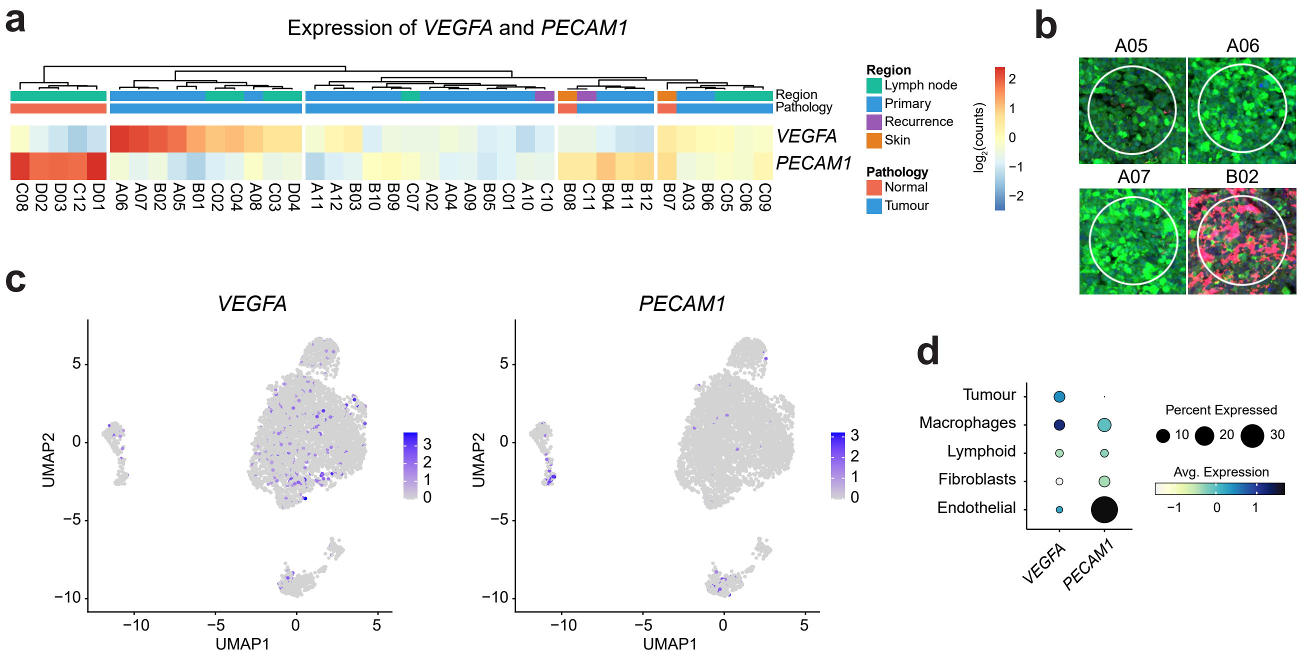
a
Expression of VEGFA and PECAM1
Region
Pathology
VEGFA
PECAM1
C08 D02 D03 C12 D01 A06 A07 B02 A05 B01 C02 C04 A08 C03 D04 A11 A12 B03 B10 B09 C07 A02 A04 A09 B05 C01 A10 C10 B08 C11 B04 B11 B12 B07 A03 B06 C05 C06 C09
Region
Lymph node
Primary
Recurrence
Skin
Pathology
Normal
Tumour
log2(counts)
2
1
0
−1
−2
b
A05
A06
A07
B02
c
VEGFA
PECAM1
UMAP2
UMAP1
5
0
−5
−10
−10
−5
0
5
3
2
1
0
d
Tumour
Macrophages
Lymphoid
Fibroblasts
Endothelial
VEGFA
PECAM1
Percent Expressed
10
20
30
Avg. Expression
−1
0
1

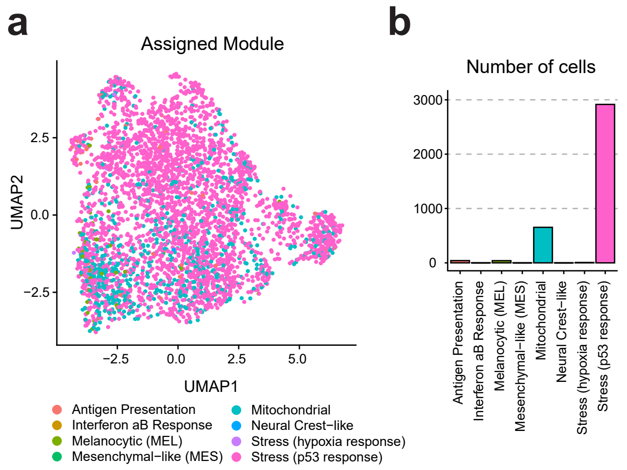
a
Assigned Module
UMAP2
2.5
0.0
−2.5
−2.5
0.0
2.5
5.0
UMAP1
Antigen Presentation
Interferon aB Response
Melanocytic (MEL)
Mesenchymal−like (MES)
Mitochondrial
Neural Crest−like
Stress (hypoxia response)
Stress (p53 response)
b
Number of cells
3000
2000
1000
0
Antigen Presentation
Interferon aB Response
Melanocytic (MEL)
Mesenchymal−like (MES)
Mitochondrial
Neural Crest−like
Stress (hypoxia response)
Stress (p53 response)

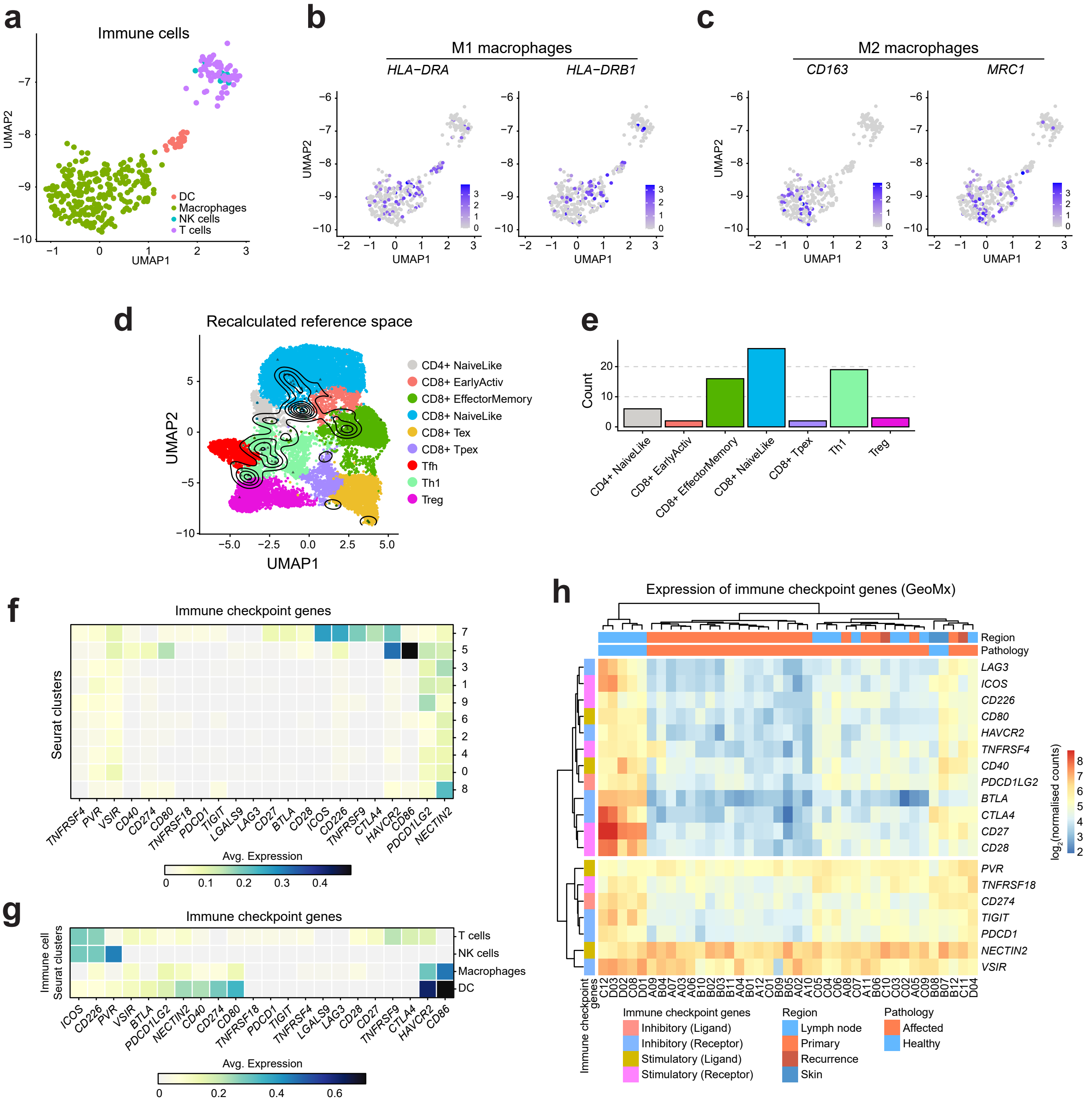
a
Immune cells
UMAP1
UMAP2
DC
Macrophages
NK cells
T cells
b
M1 macrophages
HLA−DRA
HLA−DRB1
c
M2 macrophages
CD163
MRC1
d
Recalculated reference space
UMAP1
UMAP2
CD4+ NaiveLike
CD8+ EarlyActiv
CD8+ EffectorMemory
CD8+ NaiveLike
CD8+ Tex
CD8+ Tpex
Tfh
Th1
Treg
e
Count
f
Immune checkpoint genes
Seurat clusters
Avg. Expression
TNFRSF4 PVR VSIR CD40 CD274 CD80 TNFRSF18 PDCD1 TIGIT LGALS9 LAG3 CD27 BTLA CD28 ICOS CD226 TNFRSF9 CTLA4 HAVCR2 CD86 PDCD1LG2 NECTIN2
g
Immune checkpoint genes
Immune cell Seurat clusters
T cells
NK cells
Macrophages
DC
ICOS CD226 PVR VSIR BTLA PDCD1LG2 NECTIN2 CD40 CD274 CD80 TNFRSF18 PDCD1 TIGIT TNFRSF4 LGALS9 LAG3 CD28 CD27 TNFRSF9 CTLA4 HAVCR2 CD86
Avg. Expression
h
Expression of immune checkpoint genes (GeoMx)
Region
Pathology
LAG3 ICOS CD226 CD80 HAVCR2 TNFRSF4 CD40 PDCD1LG2 BTLA CTLA4 CD27 CD28 PVR TNFRSF18 CD274 TIGIT PDCD1 NECTIN2 VSIR
log2(normalised counts)
Immune checkpoint genes
Inhibitory (Ligand)
Inhibitory (Receptor)
Stimulatory (Ligand)
Stimulatory (Receptor)
Region
Lymph node
Primary
Recurrence
Skin
Pathology
Affected
Healthy
