# Supplementary material for: Multi-modal omics analysis of a paediatric melanoma highlights mechanisms underlying treatment resistance
Source: Commun Med (Lond). 2025 Oct 30;5:448. doi: 10.1038/s43856-025-01201-1 (PMC12575648; doi:10.1038/s43856-025-01201-1)
Supplement: Supplementary file 3 — Description of Additional Supplementary Files [file 43856_2025_1201_MOESM3_ESM.pdf]

Description of Additional Supplementary Files

File name: Supplementary Data 1

Description: A list of significantly differentially expressed genes from Figures 3a and 3b
